# Retraction: Stromal interaction molecule 1 (STIM1) regulates growth, cell cycle and apoptosis of human tongue squamous carcinoma cells

**DOI:** 10.1042/BSR-2016-0519_RET

**Published:** 2023-03-02

**Keywords:** apoptosis, cell cycle, STIM1, Tca-8113 cells, tongue squamous carcinoma

This Retraction follows an Expression of Concern relating to this article previously published by Portland Press.

This article is being retracted from *Bioscience Reports* at the request of the Editor-in-Chief and the Editorial Board following receipt of a notification from a reader, alerting the Editorial Board to incorrect nucleotide sequence reagents listed. The errors in this article were identified using The Seek & Blastn tool by Labbé et al. 2019 (https://doi.org/10.1371/journal.pone.0213266).

The authors have been contacted with regards to the retraction, and have not responded to the Journal's queries and the concerns raised. Given the extent of the issues raised, the Editorial Board stand by the decision to retract the article.

